# Web-Based Telemonitoring and Delivery of Caregiver Support for Patients With Parkinson Disease After Deep Brain Stimulation: Protocol

**DOI:** 10.2196/resprot.4044

**Published:** 2015-03-06

**Authors:** Sara Marceglia, Elena Rossi, Manuela Rosa, Filippo Cogiamanian, Lorenzo Rossi, Laura Bertolasi, Alberto Vogrig, Francesco Pinciroli, Sergio Barbieri, Alberto Priori

**Affiliations:** ^1^Clinical Center for Neurostimulation, Neurotechnology, and Movement DisordersFondazione IRCCS Ca'Granda Ospedale Maggiore PoliclinicoMilanItaly; ^2^eHealthLABDepartment of Electronics, Information and BioengineeringPolytechnic University of MilanMilanItaly; ^3^Clinical Center for Neurostimulation, Neurotechnology, and Movement DisordersFondazione IRCCS Ca\'Granda Ospedale Maggiore PoliclinicoMilanItaly; ^4^Section of NeurologyDepartment of Neurological, Neuropsychological, Morphological, and Motor SciencesUniversity of VeronaVeronaItaly; ^5^Clinical Center for Neurostimulation, Neurotechnology, and Movement DisordersFondazione IRCCS Ca'Granda Ospedale Maggiore Policlinico, Milan, ItalyMilanItaly

**Keywords:** telemedicine, deep brain stimulation, Parkinson disease, delivery of health care, integrated, mobile applications

## Abstract

**Background:**

The increasing number of patients, the high costs of management, and the chronic progress of the disease that prevents patients from performing even simple daily activities make Parkinson disease (PD) a complex pathology with a high impact on society. In particular, patients implanted with deep brain stimulation (DBS) electrodes face a highly fragile stabilization period, requiring specific support at home. However, DBS patients are followed usually by untrained personnel (caregivers or family), without specific care pathways and supporting systems.

**Objective:**

This projects aims to (1) create a reference consensus guideline and a shared requirements set for the homecare and monitoring of DBS patients, (2) define a set of biomarkers that provides alarms to caregivers for continuous home monitoring, and (3) implement an information system architecture allowing communication between health care professionals and caregivers and improving the quality of care for DBS patients.

**Methods:**

The definitions of the consensus care pathway and of caregiver needs will be obtained by analyzing the current practices for patient follow-up through focus groups and structured interviews involving health care professionals, patients, and caregivers. The results of this analysis will be represented in a formal graphical model of the process of DBS patient care at home. To define the neurophysiological biomarkers to be used to raise alarms during the monitoring process, neurosignals will be acquired from DBS electrodes through a new experimental system that records while DBS is turned ON and transmits signals by radiofrequency. Motor, cognitive, and behavioral protocols will be used to study possible feedback/alarms to be provided by the system. Finally, a set of mobile apps to support the caregiver at home in managing and monitoring the patient will be developed and tested in the community of caregivers that participated in the focus groups. The set of developed apps will be connected to the already existing WebBioBank Web-based platform allowing health care professionals to manage patient electronic health records and neurophysiological signals. New modules in the WebBioBank platform will be implemented to allow integration and data exchange with mobile health apps.

**Results:**

The results of this project will provide a novel approach to long-term evaluation of patients with chronic, severe conditions in the homecare environment, based on caregiver empowerment and tailored applications developed according to consensus care pathways established by clinicians.

**Conclusions:**

The creation of a direct communication channel between health care professionals and caregivers can benefit large communities of patients and would represent a scalable experience in integrating data and information coming from a clinical setting to those in home monitoring.

## Introduction

### Background

Parkinson disease (PD) is a common neurodegenerative disorder affecting about 1% of the population over the age of 60 years [1]. PD is typically characterized by motor symptoms; however the clinical spectrum of the disease is more extensive, covering a wide range of nonmotor symptoms including cognitive and behavioral changes, sleep disorders, autonomic dysfunctions, sensory symptoms, and fatigue [2,3]. The surgical treatment of PD was reintroduced in the late 1990s with the advent of subthalamic deep brain stimulation (DBS) [4,5], and this is now an established treatment for PD patients [6-9].

Follow-up studies in PD show that after DBS surgery, motor function and performance during daily living activities improve for up to 5 to 10 years, although the initial benefit in part progressively deteriorates [10] and patients still experience clinical fluctuations that prevent them from performing simple daily activities [11]. In addition, immediately after surgery` PD patients face a highly fragile stabilization period, in which parameters are set and patients and families get used to the new situation introduced with DBS.

The long-term outcome of DBS depends on the development of unresponsive PD disturbances related to disease progression that should be properly monitored to allow early recognition and treatment [9].

Long-term PD disability involves motor and nonmotor symptoms that worsen in time. Cognitive impairment and dementia have a higher prevalence in older patients [12,13]. Dementia and hallucinations predict nursing home placement and seem to double the mortality risk especially in older PD patients [14]. Another common, bothersome nonmotor symptom is the onset of lower urinary tract symptoms [15]. Up to 68% of PD patients fall every year, with approximately 50% falling repeatedly [16]. Cognitive behavioral education and exercise training were suggested to be effective in reducing the risk of falls [17,18]. Pain affects 29% to 85% of PD patients who experience various types of pain that may fluctuate with motor symptoms as “nonmotor fluctuations” [19]; the pain improves after DBS [20].

PD progression often leads to hospitalization [21]. The most frequent causes for admission are falls, pneumonia, urinary tract infections, reduced mobility, psychiatric disorders, and mental status changes [22]. PD progression in DBS patients is monitored in scheduled follow-up clinical visits (once or twice a year) when the neurologist takes an instantaneous picture of the patient’s condition that cannot completely reflect the daily condition. At home, patients rely on a family caregiver, who is usually not trained to deal with PD progression and DBS. Often, the reference center is far from the patient's house.

Research carried out in the last 15 years shows that recordings of neuronal activity (local field potentials [LFPs]) from the implanted electrodes provide information related to the patient's state [23-34]. LFPs correlate with motor and nonmotor PD symptoms and were recently proposed as a feedback variable for new DBS systems able to adapt stimulation parameters to the patient’s state [11]. Provided that the new generation of DBS stimulators will allow LFP recordings during stimulation [11], it is likely that the analysis of neuronal activity in DBS patients can be integrated into monitoring systems to provide feedback and alarms.

These observations suggest that monitoring DBS patients more closely and continuously might be crucial to ensure a better quality of life. The use of telemedicine for monitoring PD patients has attracted a lot of attention [35-38], but no technology has been developed to support the continuous monitoring of DBS patients at home, including neurophysiological monitoring, together with an effective empowerment tool for caregivers.

### Objectives

#### Consensus Guideline and Requirements Set

The reference guideline will be developed by the multiprofessional team responsible for the patient’s care (neurologist, neurophysiologist, nurse, psychologist, physiotherapist, speech therapist) via modeling and defining the clinical care pathway of the DBS PD patient at home. In addition, the team will work with associates of the patient to establish a shared set of needs of the caregiver and patient community for the optimal management of patients with PD undergoing DBS at home.

#### Neurophysiological Biomarkers

Neurophysiological biomarkers provided by the neuronal signals recorded from deep brain electrodes can be used to directly monitor the patient's state at home. Motor, cognitive, and behavioral protocols will be used to study possible feedback/alarms to be provided by the system. These biomarkers will ground the future management of DBS PD patients at home, with the implanted stimulators being part of a system, already available as an experimental device, that will be able to record from DBS electrodes while stimulating.

#### Information System Architecture

The architecture of the information system will be created to meet the specific needs of caregivers at home by integrating the clinical care pathway of the patient in order to provide a caregiver support system connected to and sharing information with the electronic health record of the patient. The platform will be able to manage neurosignals analysis and provide alerts related to changes in biomarkers underlying changes in the patient's state. The architecture will hence be specific for home telemonitoring of DBS PD patients and empowerment of caregivers and include educational material, a direct communication channel with the specialist, and operative instructions for the recognition and management of simple alert symptoms.

## Methods

### Defining and Modeling the Process of Homecare Monitoring for DBS Patients

To achieve the definition of the consensus care pathway for PD patients with DBS implant and establish a shared set of caregiver needs, we will analyze the current management practices by involving neurologists and psychologists (maximum 15) from the DBS Study Group of the Italian Neurological Society (Società Italiana di Neurologia) and organizing a focus group dedicated to homecare management of patients. The focus group will draft a consensus clinical care pathway that will be validated by a larger group of at least 30 PD experts.

We will then prepare structured interviews for patients and caregivers to establish their main needs while at home, and we will interview patients and caregivers recruited through the Associazione Italiana Parkinson and directly through the Movement Disorders Ambulatory of both the Borgoroma Hospital and the Fondazione IRCCS Ca’Granda Ospedale Maggiore Policlinico. The expected number of patients and caregivers to be interviewed is 50.

Finally, we will involve 10 to 15 patients, together with their caregivers and reference neurologists, in a focus group dedicated to the definition of the main needs and requirements for home monitoring support systems starting from the results of the interviews.

After having completed the needs analysis for both health care professionals and patients and families, we will model through a graphical language (Unified Modeling Language [UML]) the care pathway of the PD patients with DBS at home, according to the clinical care pathway and the patient and caregiver needs.

UML is a widely used visual language for specifying, visualizing, constructing, and documenting the artifacts of software systems, as well as for business modeling and other nonsoftware applications [39,40]. A UML model is composed of diagrams modeling the static and dynamic behaviors of the system/process. The UML modeling approach is based on the decomposition of a complex system into many different objects representing the subjects/objects related through hierarchical and functional relationships and specified through properties. The methods available to each object represent all the actions that the object exposes or receives. The methods available map all the existing interactions between objects in the system. This decomposed and simplified structure allows the introduction of a formal representation and supports the verification of its validity.

### Defining Neurophysiological Biomarkers

**Figure 1:**
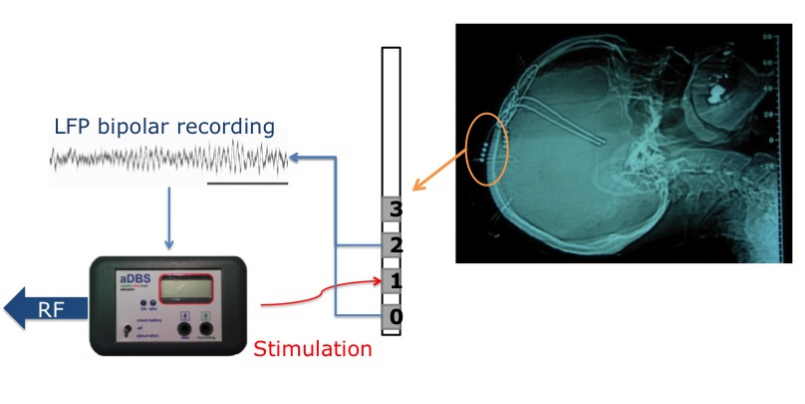
LFPs are recorded bipolarly from the DBS electrodes while the stimulation is turned ON through the aDBS device that also allows DBS parameter change and radiofrequency transmission of the recorded signals.

To define neurophysiological biomarkers (Table 1) providing feedback on the patient’s state, we will record LFPs from 30 patients affected by PD and implanted with subthalamic nucleus DBS electrodes through a new experimental system that records LFPs while DBS is ON [41] and transmits signals by radiofrequency to a recording system (Figure 1). Recordings will take place 2 to 3 days after the surgery for electrode implantation and will record continuously for 3 hours. All of the experimental sessions will be videorecorded to allow further offline reassessment of the patient’s state by an experienced neurologist in order to detect motor fluctuations and other symptoms that may require feedback to the caregiver or to the reference neurologist.

LFPs will be bipolarly recorded through DBS electrodes (Medtronic 3389) implanted in the subthalamic nucleus. The DBS electrode has four cylindrical contacts (0, 1, 2, and 3 beginning from the most caudal). Two contacts will be used for recording and one for DBS delivery. LFPs will be recorded through a new system (aDBS, Newronika srl) that allows LFP recording with DBS ON [41]; control of DBS parameters, manual and automatic, through ad hoc algorithms; and radiofrequency transmission of recorded signals.

LFP analysis will be conducted offline with ad hoc programs stored on the WebBioBank system for clinical data collection. These Matlab-based programs allow signal preprocessing and analysis and were previously used for other LFP analyses [25,26,28,33].

Because LFPs represent the activity of large populations of neurons, the analysis will be carried out in the frequency domain. Spectral analysis (power spectrum), cross-spectral analysis (coherence), parametric and nonparametric time-frequency analysis, and bispectral analysis (bicoherence) will be applied to extract biomarkers. Parametric and nonparametric tests will be used to evaluate the intrasubjective changes of LFP biomarkers in different conditions and during different tasks.

**Table 1:** Protocols for defining neurophysiological biomarkers.

LFP recording		DBS
After 12 hours withdrawal of antiparkinsonian therapy:		
	Rest (with the patient lying in an armchair or on a bed)	Off
	Rest (with the patient lying in an armchair or on a bed)	On
	Movement (during self-paced repetitive movement of one arm/leg, during cued movements of the limbs, and during spontaneous walking around the room)	Off
	Movement (during self-paced repetitive movement of one arm/leg, during cued movements of the limbs, and during spontaneous walking around the room)	On
	Emotional and decision-making tasks (during an emotional task—patient will be shown pictures/movies that elicit emotional response—and during a decision-making task—patient must choose an answer to moral and neutral questions)	Off
	Emotional and decision-making tasks (during an emotional task—patient will be shown pictures/movies that elicit emotional response—and during a decision-making task—patient must choose an answer to moral and neutral questions)	On
After the administration of a clinically effective dose of levodopa:		
	Rest (with the patient lying in an armchair or on a bed)	Off
	Rest (with the patient lying in an armchair or on a bed)	On
	Movement (during self-paced repetitive movement of one arm/leg, during cued movements of the limbs, and during spontaneous walking around the room)	Off
	Movement (during self-paced repetitive movement of one arm/leg, during cued movements of the limbs, and during spontaneous walking around the room)	On
	Emotional and decision-making tasks (during an emotional task—patient will be shown pictures/movies that elicit emotional response—and during a decision-making task—patient must choose an answer to moral and neutral questions)	Off
	Emotional and decision-making tasks (during an emotional task—patient will be shown pictures/movies that elicit emotional response—and during a decision-making task—patient must choose an answer to moral and neutral questions)	On

### Defining the Architecture of the Integrated Telecare System

The implementation of the system architecture will be based on the extension of the already-existing WebBioBank system for the management of electronic health records (EHR) of PD patients with DBS electrode implants, integrated with signal analysis [42]. The system will be extended with a set of mobile apps to support the caregiver in managing and monitoring the patient at home.

**Figure 2:**
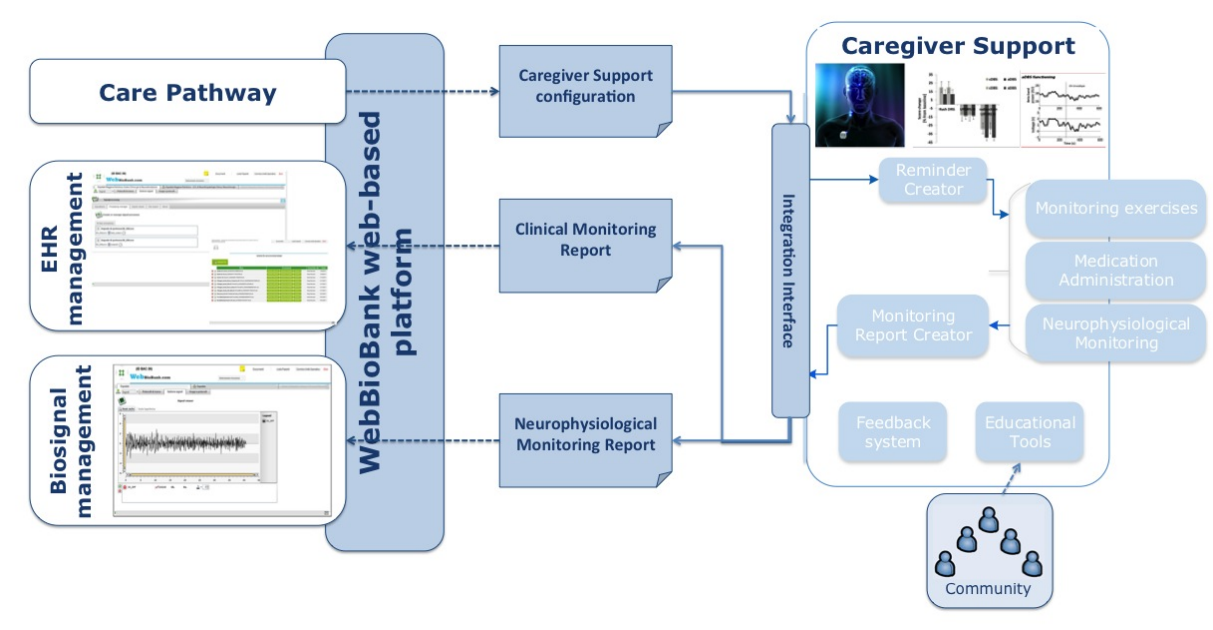
Expected architecture of the prototype system. The WebBioBank Web-based platform supports the information exchange between the Care Pathway module that defines the configuration of the homecare monitoring/treatments and the Caregiver Support module, a mobile app dedicated to the caregiver.

The architecture will consist of two modules, Care Pathway and Caregiver Support, which are both connected to the WebBioBank Web-based platform (Figure 2). The Care Pathway module will be a part of the WebBioBank EHR system and will be devoted to supporting the continuous monitoring of the disease progression and updating of home treatments, customized for each patient. The Caregiver Support module will be designed to provide the tools and information the patient and caregiver need for the proper implementation of the Care Pathway, according to the needs and requirements defined in the first phase of the project. A set of mobile apps will be made available to patients and caregivers to support symptom interpretation, emergency management, and routine daily activities and allow information exchange with the reference neurologist. A mobile device, such as a tablet, will be provided to patients who do not have a personal one.

The system will be implemented and preliminary tested (alpha test) on the same patients involved in the focus groups previously described. System validation will be not part of this project.

## Results

The project is supported by preliminary observations showing that PD patient and caregiver associations are already active in asking for more effective communication with their care team and reliable education sources for the management of patients in homecare. Also, our previous studies show that neurophysiological signals from DBS electrodes provide information on PD motor and nonmotor symptoms [11,34], and we have already implemented a platform for the management and analysis of biopotentials recorded from DBS electrodes, integrated with WebBioBank [42].

We expect at the end of the project to obtain a reference consensus guideline and a shared requirements set for the home monitoring and care of patients with PD treated with DBS that, combined with the set of biomarkers that will provide alarms/feedback to caregivers and health care professionals regarding the patient’s state, will constitute the implementation of continuous home monitoring for DBS patients. The implementation of a prototype information system architecture allowing communication between health care professionals and caregivers will support the improvement of the quality of care for PD patients treated with DBS.

## Discussion

Patients with PD undergoing DBS face a highly fragile stabilization period and require specific support at home. At present, no system for DBS home monitoring is available. Our project, introducing effective direct and continuous monitoring of patients at home, would help not only to assess PD progression but also to make the patients and their families central actors in the care process.

More specifically, the definition of consensus care pathways for patient home monitoring, the definition of neurophysiological biomarkers from signals recorded through DBS electrode, and the in-depth analysis of caregiver needs will ground the development of supporting tools and telemonitoring of DBS PD patients at home. Properly integrated with patient EHRs, these tools will improve the quality of life for DBS PD patients by supporting caregivers, optimizing patient continuous monitoring, and providing alerts to professionals and caregivers about disease worsening or reprogramming needs.

The project results will contribute to the integration of the caregiver role in the care pathway of the patient through a direct communication channel between health care professionals and caregivers by means of an enhanced and easily accessible mobile app system for DBS patient home monitoring. In the project, we will also address the issue of ongoing support to caregivers through dedicated apps facilitating their education and interaction with local resources. Also, the definitions by clinicians of consensus care pathways and personalized care plans may produce better resource management for the national health care service and may improve, in terms of effectiveness and efficiency, the management of this costly neurodegenerative chronic disease.

The experience in integrating data and information coming from a clinical setting to those in home monitoring can be scalable to other conditions involving patients with chronic diseases in the homecare environment, and tailored apps can be developed according to consensus care pathways established by clinicians.

## Abbreviations

PD: Parkinson disease
DBS: deep brain stimulation
LFP: local field potential
UML: Unified Modeling Language
EHR: electronic health record
